# Innovative use of *Dendrobium devonianum* paxt stem residue as a bioactive feed additive: enhancing roasted chicken flavor and nutritional quality through metabolomics and molecular docking

**DOI:** 10.3389/fvets.2026.1822882

**Published:** 2026-05-21

**Authors:** Zhen Zhang, Dongying Zhang, Zuhong Li, Yuwei Guo, Chengxu Liu, Cunchao Zhao, Ya Wang

**Affiliations:** 1 College of Food Science and Technology, Yunnan Agricultural University, Kunming, China; 2 College of Science, Yunnan Agricultural University, Kunming, China; 3 Yunnan Pinhutang Biotechnology Co., Ltd, Baoshan, China; 4 Yunnan Plateau Characteristic Agricultural Industry Research Institute, Yunnan Agricultural University, Kunming, China; 5 Yunnan Key Laboratory of Precision Nutrition and Personalized Food Manufacturing, Yunnan Agricultural University, Kunming, China; 6 Key Laboratory of Development and Utilization of Food and Medicinal Resources, Ministry of Education, Kunming, China

**Keywords:** bioactive feed additives, dendrobium devonianum paxt stem residue, meat quality, metabolomics, poultry nutrition, sustainable feed ingredients

## Abstract

**Introduction:**

This study evaluated the effects of *Dendrobium devonianum* Paxt stem residue (DPSR) and its fermented form (FDPSR) as dietary ingredients on broiler growth performance and roasted chicken quality.

**Methods:**

A total of 63 white-feathered broilers were assigned to three dietary treatments for 45 days: CK (basal diet), DPSR (basal diet supplemented with 10% DPSR), and FDPSR (basal diet supplemented with 10% FDPSR), with three replicate pens per treatment and seven birds per pen. Roasted chicken breast was analyzed by texture profile analysis, oxidation-related assays, E-nose, E-tongue, LC-MS, HS-SPME-GC-MS, and molecular docking simulation analysis.

**Results:**

During days 1–21, both DPSR and FDPSR reduced average daily gain to 24.70 and 26.60 g/bird/day, respectively, compared with 33.67 g/bird/day in the CK group (*p* < 0.05). Furthermore, feed conversion ratios increased feed to 1.66 and 1.54 for DPSR and FDPSR, respectively, compared with 1.27 in the CK group (p < 0.05). In contrast, no significant differences were observed among treatments during days 22-45 or over the entire 45-day feeding period. In roasted chicken, DPSR improved selected textural attributes, whereas FDPSR was more strongly associated with flavor-related variations. Both treatments significantly reduced malondialdehyde content after roasting (*p* < 0.05). Metabolomics identified 52 differential metabolites, with aldehydes being markedly higher in the FDPSR group (67.81%) than in the CK (43.56%) and DPSR (40.11%) groups. Molecular docking simulation suggested potentially favorable interactions between representative aroma compounds and selected olfactory receptors.

**Discussion:**

Overall, DPSR and FDPSR altered the texture, oxidative stability, and flavor profiles of roasted chicken, with FDPSR demonstrating a more pronounced effect on flavor-related metabolites and volatile compounds. These findings provide preliminary support for the use of DPSR-based ingredients in poultry production, although further validation is required.

## 1 Introduction

*Dendrobium devonianum Paxt*, a medicinal and edible plant that has attracted growing attention in recent years, is rich in proteins, vitamins, phytosterols, and flavonoids, making it highly valuable for pharmaceutical and functional food applications (1). Its processing byproduct, *Dendrobium devonianum* Paxt stem residue (DPSR), contains substantial amounts of dietary fiber (DF), polysaccharides, and flavonoids, and therefore may have potential as a functional feed ingredient. DF, recognized as the “seventh nutrient,” has been shown to regulate gut microbiota, improve lipid metabolism, and reduce the risk of cardiovascular diseases (2–4). For example, Leontowicz et al. (5) reported that dietary fibers derived from beet pulp and apple pomace significantly improved lipid metabolism in hypercholesterolemic rats. In livestock and poultry production, dietary fiber supplementation not only enhances animal welfare—by increasing satiety and reducing stereotypic behaviors—but also improves growth performance (6). Similarly, Li et al. (7) found that a total dietary fiber level of 23.4% yielded the greatest weight gain in juvenile catfish. However, research on the effects of dietary fiber on meat quality—particularly flavor and sensory attributes—remains limited.

Chicken is widely favored by consumers for its high protein and low fat content (8), with roasted chicken being a leading market product due to its distinctive flavor and texture. Current research on roasted chicken has primarily focused on processing techniques, including the effects of different heating temperatures on common component levels (9), microbial dynamics during storage (10), and the inhibitory effects of additives on heterocyclic amine formation (11). In modern broiler production, soybean meal is commonly used as the primary protein source in feed formulations to improve meat quality. However, its heavy reliance on imports and price volatility pose challenges to the sustainable development of the poultry industry (12, 13). Alternative protein sources such as rapeseed meal and cottonseed meal are readily available but may negatively affect poultry growth (14). Solid-state fermentation (SSF) has been shown to enhance the nutritional value of unconventional protein sources (15). For DPSR, fermentation may be particularly beneficial, as the raw material is rich in dietary fiber and other plant-derived components that may limit nutrient accessibility and utilization in poultry. Fermentation has the potential to partially degrade complex structural components, liberate small bioactive molecules, and modify flavor-related precursors, thereby improving the functional value of DPSR as a feed ingredient. Therefore, comparing DPSR with its fermented form (FDPSR) is necessary to determine whether fermentation can further enhance its application potential in broiler diets. For example, Lei et al. (16) demonstrated that fermented feed can significantly improve growth performance and lean meat yield in pigs, though its application in broiler diets remains largely unexplored. Meat quality traits such as tenderness, juiciness, and flavor are key determinants of consumer preference (17). Gál et al. (18) compared the effects of different cooking methods on chicken quality using texture analysis and sensory evaluation. In recent years, advanced analytical techniques have been increasingly applied in meat research: electronic nose (E-nose) and headspace solid-phase microextraction gas chromatography mass spectrometry (HS-SPME-GC-MS) for volatile compound profiling, and electronic tongue (E-tongue) and liquid chromatography-mass spectrometry (LC-MS) for non-volatile metabolite analysis, offering greater efficiency and precision in flavor and quality assessment (19).

This study aims to investigate the effects of dietary supplementation with DPSR and FDPSR on the quality and flavor of roasted chicken. It was hypothesized that both DPSR and FDPSR could modulate roasted chicken quality and flavor, and that fermentation might further enhance the application value of DPSR as a sustainable feed ingredient. Accordingly, DPSR and FDPSR were compared using metabolomics, molecular docking simulation, and instrumental sensory analyses to assess whether fermentation could enhance the feeding value of DPSR in broiler diets.

## 2 Materials and methods

### 2.1 Animals, husbandry, and sample preparation

A total of 63 white-feathered broilers were randomly assigned to three dietary treatments: CK (basal diet), DPSR (basal diet supplemented with 10% DPSR), and FDPSR (basal diet supplemented with 10% FDPSR). The 10% inclusion level was selected as a literature-informed exploratory dose to provide an initial comparison between DPSR and FDPSR in broiler diets. However, because no dose-response design was included, the optimal supplementation level remains to be determined (20). The basal diet contained 58.50% corn, 29.60% soybean meal, 3.00% soybean oil, 5.00% peanut vine, 0.22% salt, 0.1% threonine, 0.18% methionine, 1.40% dicalcium phosphate, and 2.00% premix. The DPSR was purchased from Yunnan Pinhutang Biotechnology Co., Ltd. (Baoshan, China). For the FDPSR diet, dried DPSR was mixed with purified water at a 1:10 ratio, inoculated with Lactiplantibacillus plantarum LB-8 and mixed Lactiplantibacillus ABY-8 (≥11 × 011 CFU/g, Chr. Hansen Co., Ltd.) at a 1:2000 ratio, and fermented at 38–42°C for 72 h. After fermentation, the mixture was freeze-dried and incorporated into the standard diet at a 1:9 ratio. Each treatment consisted of three replicate pens, with seven birds per pen. The feeding trial lasted for 45 days. The chicken house was cleaned and disinfected 1 week before the experiment. Birds were housed in a semi-enclosed poultry house with manually controlled temperature, humidity, and ventilation. The initial temperature was maintained at 32°C and then gradually reduced by 2–3°C per week until it reached 20°C. Continuous lighting was provided throughout the experimental period. Feed was supplied using plastic feeders, and drinking water was provided in plastic buckets and refreshed once daily. Body weight, average daily feed intake (ADFI), average daily gain (ADG), and feed conversion ratio (FCR) were recorded weekly on a pen basis. At 7 days of age, birds were vaccinated against Newcastle disease by eye drop administration, and at 12 days of age, they were vaccinated against infectious bursal disease *via* drinking water. Standard management procedures were strictly followed throughout the experiment to maintain good hygiene and health status in the poultry house. For diet preparation, 1 kg of DPSR or FDPSR was mixed with 9 kg of basal diet in a container and thoroughly homogenized by manual stirring before feeding. The CK group received the basal diet without DPSR or FDPSR supplementation.

After 45 days of feeding, one bird from each pen whose body weight was closest to the mean body weight of that pen was selected for slaughter and subsequent analyses. Therefore, three independent biological replicates per treatment group (*n* = 3) were used for roasted meat quality evaluation, LC-MS, HS-SPME-GC-MS, E-nose, and E-tongue analyses. The right-side breast muscles collected from the selected birds were cut into 3 cm × 3 cm × 3 cm cubes following the orientation of the muscle fibers. The meat pieces were then evenly arranged on a baking tray at 2 cm intervals and roasted in a preheated electric oven (Xinmai Machinery Co., Ltd., Wuxi, Jiangsu, China) at 180°C for 18 min. This preparation procedure was repeated three times to standardize sample size, lean layer consistency, and muscle fiber orientation for subsequent analyses.

### 2.2 Growth performance calculation

Body weight was recorded weekly for each bird, and the average body weight of each pen was calculated. Feed intake was recorded weekly on a pen basis as the difference between feed offered and feed refused. Since each pen contained seven birds and no mortality occurred during the experimental period, average daily feed intake (ADFI, g/bird/day) was calculated as weekly feed intake divided by 49 bird-days. Average daily gain (ADG, g/bird/day) was calculated from the weekly increase in pen mean body weight divided by 7 days. Feed conversion ratio (FCR) was calculated as total feed intake divided by total body weight gain for each pen. Pen was considered the experimental unit for growth performance analysis.

### 2.3 Color measurement

Color measurement and calibration were conducted following the method described by Wang et al. (21). A colorimeter (CR- 410, Minolta, Japan) was used to measure each sample in triplicate. Lightness (L^*^), redness/greenness (a^*^), and yellowness/blueness (b^*^) were recorded as per standard colorimetric coordinates (22). The total color difference (ΔE) and browning index (BI) between raw and roasted chicken breast surfaces were calculated according to the following formulas (23, 24):


$$\begin{array}{l} \text{Δ}E={\left[{\left(L^{*}-L_{0}^{*}\right)}^{2}+{\left(a^{*}-a_{0}^{*}\right)}^{2}+{\left(b^{*}-b_{0}^{*}\right)}^{2}\right]}^{1/2} \end{array}$$ (1)

Where ${\text{L}}_{0}^{*}$, ${\text{a}}_{0}^{*}$, ${\text{b}}_{0}^{*}$ and L^*^, a^*^, b^*^ are the color parameters of raw meat and roasted ones, respectively.


$$\begin{array}{l} \text{BI}\left(Browningindex\right)=\left[100\times \left(x-0.312\right)\right]/0.172\; \end{array}$$ (2)

Where x = (a^*^ + 1.75 L^*^) / (5.645 L^*^ + a^*^ - 3.012b^*^)

### 2.4 Texture analysis

Cubic samples (10 mm × 10 mm × 10 mm) excised from the roasted chicken breast were subjected to TPA following the procedure of Zheng et al. (25), with slight modifications. TPA was conducted using a texture analyzer (TMS-TOUCH, FTC, USA). A cylindrical probe compressed the samples to a depth of 5 mm at a speed of 1 mm/s, with a trigger force set at 0.1 N and three compression cycles. Texture parameters were extracted using Texture Pro CT software.

### 2.5 SEM and LF-NMR analysis

The microstructure of roasted chicken was examined using SEM, following the method described by Wang et al. (26). Roasted chicken slices were immersed in 2.5% glutaraldehyde solution, rinsed with 0.1 mol/L phosphate-buffered saline (PBS) for 5 min, and dehydrated through a graded ethanol series. The samples were frozen at −80 °C and subsequently freeze-dried using a freeze dryer (Alpha 1–4, Martin Christ, Osterode, Germany) for 72 h. After sputter-coating with gold, the microstructure was observed using a scanning electron microscope.

In the NMR analysis software, the Carr-Purcell-Meiboom-Gill (CPMG) pulse sequence was applied. The 90° hard pulse width (P1) and 180° hard pulse width (P2) were set to 12 μs and 23.52 μs, respectively. Other parameters included a waiting time (TW) of 3,500 ms, number of echoes (NECH) of 18,000, number of scans (NS) of 4, and a receiver bandwidth (SW) of 250 kHz. Finally, 100,000 iterations of inversion were performed, and relaxation curves were generated using NMR Analysis Software Ver 4.0 (Niumag Analytical Instruments Co., Ltd., Suzhou, China).

### 2.6 Determination of total sulfhydryl groups, protein carbonyls, and malondialdehyde (MDA) contents

The total sulfhydryl (–SH) content and protein carbonyl content of roasted chicken were determined using commercial assay kits (AKAO010M and AKAO007M; Boxbio, Beijing, China) according to the manufacturer's protocols. MDA content was quantified using the thiobarbituric acid (TBA) method with a commercial kit (Jiancheng Bioengineering Institute, Nanjing, China), based on absorbance at 532 nm.

### 2.7 E-nose and E-tongue analysis

A portable PEN3 E-nose (AIRSENSE, Schwerin, Germany) was employed to detect volatile compounds released from roasted chicken samples. The sensor array configuration of the PEN3 device followed the setup described by Bai et al. (27). Briefly, 2 g of roasted chicken sample was sealed in a vial and incubated in a water bath at 55 °C. Prior to measurement, the sensor chamber was purged with clean air. During analysis, the gas flow rate was maintained at 300 mL/min, with a sample equilibration time of 10 s. The total detection time and flushing time were set at 100 s and 300 s, respectively (28).

Taste attributes of roasted chicken were evaluated using an SA402B E-tongue (INSENT, Tokyo, Japan). Twenty grams of roasted chicken were homogenized with 100 mL of distilled water for 1 min using a disperser (PD500-TP, Prima Ltd., London, UK). The homogenate was centrifuged at 10,000 × g for 10 min, and the supernatant was filtered through a 0.45 μm membrane. The resulting filtrate was used for E-tongue analysis (29).

### 2.8 LC-MS analysis

Based on the method reported by Yu et al. (30), LC-MS analysis of roasted chicken samples was conducted using a UHPLC system (1290, Agilent Technologies, Santa Clara, CA, USA) coupled with a Q Exactive Orbitrap mass spectrometer (Thermo Fisher Scientific, Waltham, MA, USA). Chromatographic separation was achieved using a UPLC HSS T3 column (1.8 μm, 2.1 mm × 100 mm, Waters). The mobile phases consisted of 0.1% formic acid in water (A) and acetonitrile (B) for positive ion mode, and 5 mM ammonium acetate in water (A) and acetonitrile (B) for negative ion mode. Elution was performed at a flow rate of 0.5 mL/min with an injection volume of 1 μL. Mass spectrometric data were continuously acquired in both positive and negative electrospray ionization (ESI) modes using Xcalibur software (version 4.0.27; Thermo Fisher Scientific). Raw data were processed using ProteoWizard and the XCMS package in R.

### 2.9 HS-SPME-GC-MS analysis

Volatile compounds in minced roasted chicken were analyzed using a gas chromatograph (Agilent 8890A, Agilent Technologies, Palo Alto, CA, USA) coupled with a Pegasus BT 4D mass spectrometer (LECO, St. Joseph, MI, USA). Separation was performed on a DB-HeavyWax capillary column (30 m × 250 μm × 0.5 μm; Agilent, USA). High-purity helium served as the carrier gas at a constant flow rate of 1.0 mL/min. The initial oven temperature was set at 50 °C for 2 min, then ramped to 230 °C at 5 °C/min and held for 5 min. The temperatures of the transfer line and ion source were maintained at 250 °C. Data were acquired at 10 spectra/s with an electron ionization energy of 70 eV. The detector voltage was set at 1,960 V, and the mass scan range was m/z 35–550 (31).

### 2.10 Molecular docking simulation analysis

Molecular docking simulation was performed using AutoDockTools version 1.5.6 (Scripps Research Institute, USA). Six representative olfactory receptors (OR1A1, OR1G1, OR2W1, OR5M3, OR7D4, and OR8D1) and five key volatile compounds (2-isooctanone, decanal, ethylbenzene, n-tetradecane, and n-tridecane) were selected for *in silico* interaction analysis. The three-dimensional structures of both receptors and ligands were retrieved in PDB format from AlphaFold. Prior to docking, all structures were preprocessed in AutoDockTools, including hydrogen addition and removal of water molecules. Docking was performed based on predicted binding pockets, and the most favorable binding conformations were selected. The receptor–ligand complexes were visualized in PyMOL (DeLano Scientific LLC, USA) to illustrate potential binding patterns. Protein-ligand interactions were further analyzed using the Protein–Ligand Interaction Profiler (PLIP) web platform.

### 2.11 Statistical analysis

For postmortem meat quality, LC-MS, HS-SPME-GC-MS, E-nose, and E-tongue analyses, the experimental unit was one bird selected from each independent pen, resulting in three biological replicates per treatment group (*n* = 3). All data were expressed as mean ± standard deviation (SD). One-way analysis of variance (ANOVA) was performed using IBM SPSS Statistics 27 (IBM Corp., Armonk, NY, USA), followed by Tukey's *post hoc* test to determine significant differences among groups at *p* < 0.05. Spearman correlation analysis was conducted using Origin 2024 (OriginLab Corporation, Northampton, MA, USA). Multivariate analyses, including PCA and OPLS-DA, were used primarily for exploratory pattern recognition and candidate marker screening. Given the limited number of biological replicates, the corresponding metabolomics and flavor-related multivariate results were interpreted cautiously.

## 3 Results

### 3.1 Growth performance of broilers

**Figure 1:**
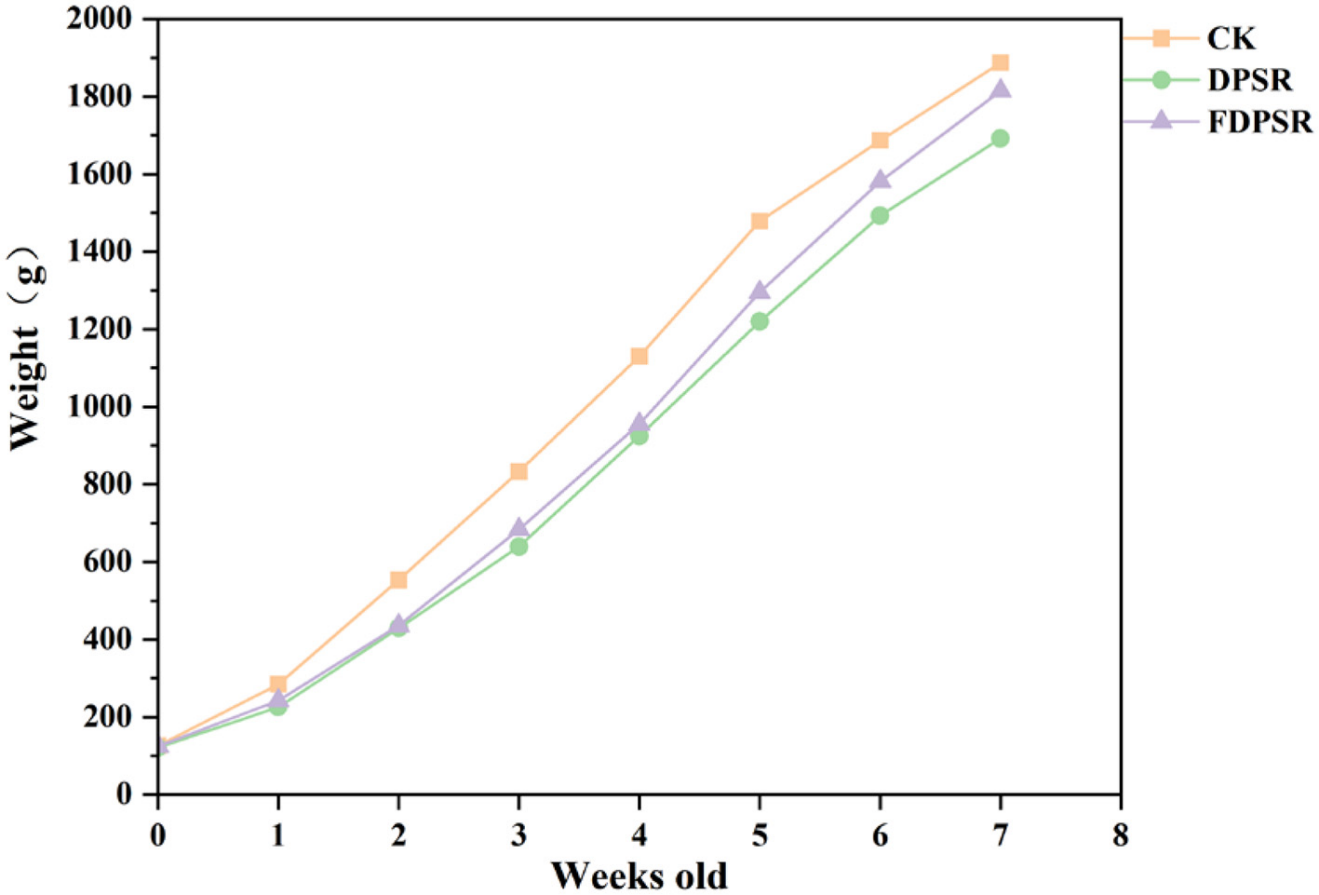
Weekly body weight changes of broilers fed diets supplemented with DPSR or FDPSR during the 45-day feeding trial.

Dietary supplementation with DPSR and FDPSR significantly affected broiler growth performance during the starter phase (days1–21; Table 1 and Figure 1). Compared with the CK group, both the DPSR and FDPSR groups showed significantly lower ADG, while no significant difference was observed in ADFI. In addition, FCR was significantly higher in the DPSR and FDPSR groups than in the CK group, indicating reduced feed utilization efficiency during the early growth stage. Specifically, ADG decreased from 33.67 g/bird/day in the CK group to 24.70 and 26.60 g/bird/day in the DPSR and FDPSR groups, respectively, whereas FCR increased from 1.27 in the CK group to 1.66 and 1.54 in the DPSR and FDPSR groups, respectively.

**Table 1:** Growth performance of broilers fed diets supplemented with DPSR or FDPSR during different feeding periods.

Item	Period	CK	DPSR	FDPSR
ADG (g/bird/day)	1–21 d	33.67 ± 0.55^a^	24.70 ± 1.65^b^	26.60 ± 0.98^b^
ADG (g/bird/day)	22–45 d	43.92 ± 6.94^a^	43.86 ± 2.71^a^	47.11 ± 5.78^a^
ADG (g/bird/day)	1–45 d	39.13 ± 3.84^a^	34.92 ± 1.72^a^	37.54 ± 3.49^a^
ADFI (g/bird/day)	1–21 d	42.66 ± 0.83^a^	41.09 ± 3.94^a^	40.95 ± 0.74^a^
ADFI (g/bird/day)	22–45 d	174.10 ± 2.30^a^	164.86 ± 21.27^a^	166.13 ± 9.93^a^
ADFI (g/bird/day)	1–45 d	101.16 ± 0.75^a^	96.11 ± 11.76^a^	96.64 ± 4.85^a^
FCR	1–21 d	1.27 ± 0.01^c^	1.66 ± 0.06^a^	1.54 ± 0.09^b^
FCR	22–45 d	3.53 ± 0.55^a^	3.29 ± 0.39^a^	3.12 ± 0.45^a^
FCR	1–45 d	2.60 ± 0.26^a^	2.75 ± 0.25^a^	2.59 ± 0.31^a^

Data are expressed as mean ± SD, with pen as the experimental unit (*n* = 3 pens per treatment). Different lowercase letters within the same row indicate significant differences among groups (*p* < 0.05).

During the grower-finisher phase (days 22–45), no significant differences were detected among the three groups in ADG, ADFI, or FCR. Likewise, when the entire 45-day experimental period was considered, all growth performance parameters remained statistically comparable among treatments. Although the FDPSR group showed numerically higher ADG and lower FCR than the DPSR group during days 22–45, these differences were not significant. Overall, these results suggest that dietary inclusion of DPSR or FDPSR may temporarily reduce growth efficiency during the early feeding stage, but this effect did not persist into the later growth period and did not significantly alter overall performance during the 45-day trial.

### 3.2 Effects of dietary supplementation on the pH and color attributes of roasted chicken

Prior to consumption, the color and appearance of roasted chicken are key indicators of quality (32). As shown in Table 2, the addition of DPSR and FDPSR to the diet had no significant effect on the pH of roasted chicken breast. However, both treatments resulted in a decrease in L^*^ values compared to the CK group. This reduction in L^*^ is likely due to the decreased water-holding capacity during roasting, which accelerates moisture evaporation and dehydration, leading to a darker color (33). The FDPSR group exhibited the highest ΔE and browning index (BI) values, suggesting more pronounced color changes and a stronger Maillard reaction during roasting.

**Table 2:** Color difference and pH analysis of chicken meat.

Item	CK	DPSR	FDPSR
L^*^	25.80 ± 44.39^a^	24.81 ± 5.01^a^	19.83 ± 0.88^a^
a^*^	5.94 ± 1.29^a^	5.82 ± 1.02^a^	5.78 ± 0.62^a^
b^*^	11.62 ± 1.72^a^	11.46 ± 2.37^a^	8.98 ± 0.46^a^
Δ*E*	3.11	2.19	8.87
*BI*	73.39	75.68	78.18
pH	6.550 ± 0.03^a^	6.58 ± 0.01^a^	6.59 ± 0.02^a^

Each the sample underwent three replications, and data were presented as mean values ± standard deviation. Different lowercase letters of superscript (a, b and c, *p* < 0.05) denote levels of compounds with statistically significant differences determined by one-way ANOVA (Duncan's multiple comparison test). The asterisk in L^*^, a^*^, and b^*^ is part of the standard colorimetric notation and does not indicate statistical significance.

### 3.3 Texture profile analysis of roasted chicken breast

TPA is widely used to characterize the textural properties of food products, which are critical indicators of overall quality and are directly associated with consumer preference (34). A smooth and elastic texture is a distinctive feature of cooked chicken, which may be attributed to its high total collagen content but low soluble collagen proportion (35). As shown in Table 3, the addition of FDPSR to the diet resulted in a reduction in both springiness and hardness of the roasted chicken breast. Apart from hardness, no significant differences in other texture parameters were observed between the FDPSR and CK groups, suggesting that FDPSR had a limited impact on overall textural quality. In contrast, the DPSR group exhibited significantly lower hardness and chewiness compared to the CK group, while springiness and adhesiveness were markedly increased. Adhesiveness reflects the degree of stickiness perceived during chewing and is an important indicator of palatability (36). These findings imply that the addition of DPSR improved the textural acceptability of roasted chicken breast.

**Table 3:** Texture analysis of chicken meat.

Item	CK	DPSR	FDPSR
Hardness (N)	8.71 ± 1.18^a^	5.64 ± 1.12^b^	6.53 ± 0.12^b^
Adhesiveness (N.mm)	0.05 ± 0.01^a^	0.10 ± 0.04^a^	0.06 ± 0.02^a^
Cohesiveness (Ratio)	0.47 ± 0.06^a^	0.57 ± 0.06^a^	0.57 ± 0.06^a^
Springiness (mm)	3.60 ± 0.35^a^	3.74 ± 0.17^a^	3.21 ± 0.24^a^
Gumminess (N)	2.67 ± 0.87^b^	4.92 ± 0.50^a^	3.89 ± 0.44^ab^
Chewiness (mj)	17.83 ± 3.46^a^	10.10 ± 3.74^b^	12.50 ± 1.41^ab^

Each the sample underwent three replications, and data were presented as mean values ± standard deviation. Different lowercase letters of superscript (a, b and ab, *p* < 0.05) denote levels of compounds with statistically significant differences determined by one-way ANOVA (Duncan's multiple comparison test).

### 3.4 Microstructural and water distribution analysis of roasted chicken breast by SEM and LF-NMR

**Figure 2:**
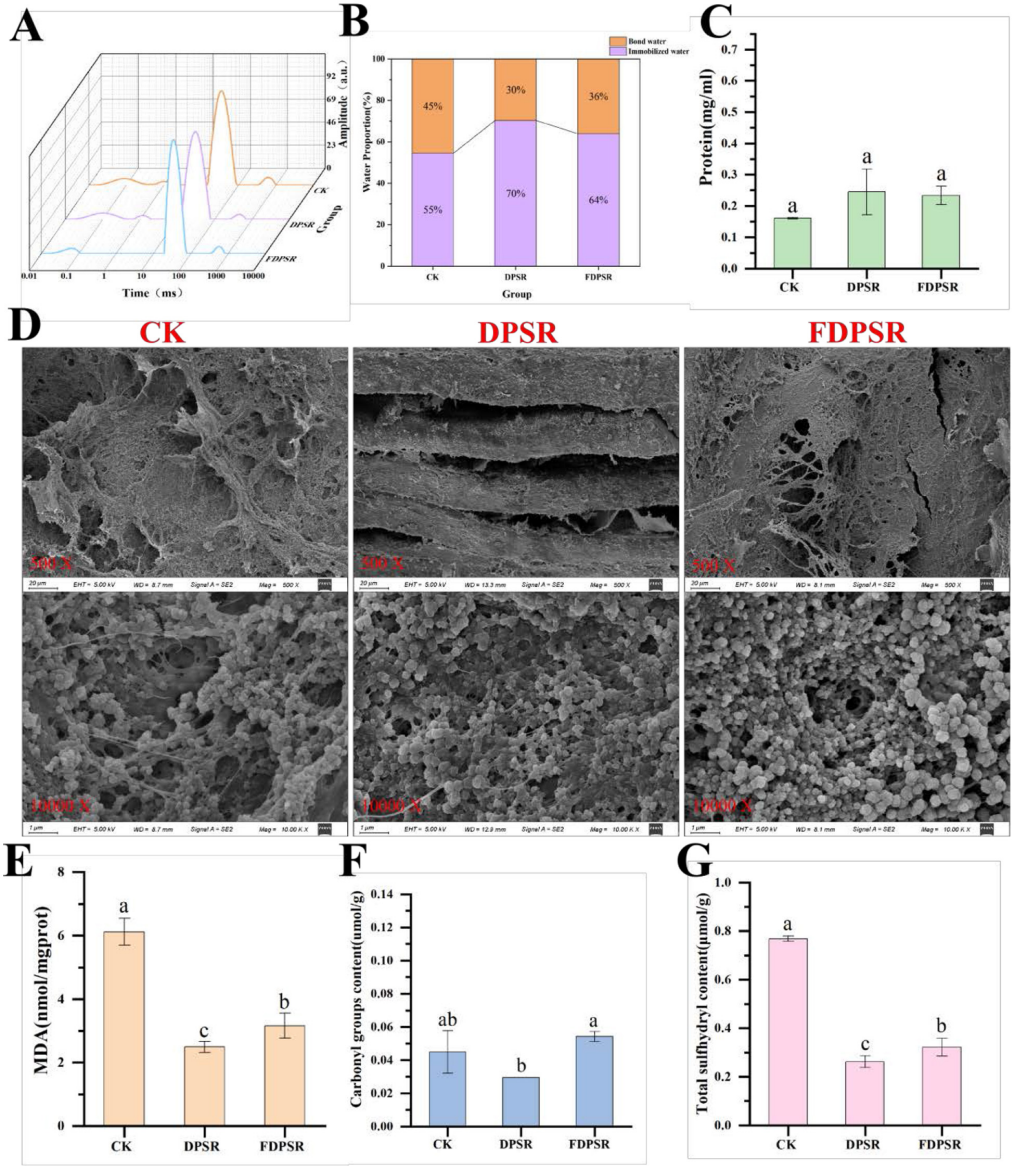
**(A, B)** Water distribution in chicken meat. **(C)** Protein concentration. **(D)** Analysis of scanning electron microscopy (SEM). **(E)** Malondialdehyde content. **(F)** Protein carbonyl content. **(G)** Total sulfhydryl content.

As shown in Figure 2D, subtle differences in microstructure were observed between the CK group and the DPSR/FDPSR groups. In both DPSR and FDPSR samples, visible gaps were observed between muscle fibers, whereas the CK group showed a relatively more compact structure. These structural differences may reflect treatment-related variation in muscle morphology after roasting (37); however, SEM in the present study provided morphological observations only and did not directly characterize the internal structural basis of water retention. LF-NMR transverse relaxation analysis was used to evaluate water mobility in roasted chicken samples. The T2 relaxation time distributions (Figure 2A) revealed three water populations: T2b (0–10 ms), corresponding to bound water; T21 (10-100 ms), representing immobilized water; and T22 (100–1000 ms), corresponding to free water (38). As shown in Figure 2B, both DPSR and FDPSR groups exhibited lower proportions of bound water and higher proportions of immobilized water than the CK group. These results indicate that dietary supplementation with DPSR or FDPSR was associated with changes in water distribution and mobility in roasted chicken breast. However, because bound water decreased while immobilized water increased, these LF-NMR results do not directly support a simple interpretation of improved water-holding capacity.

### 3.5 Antioxidant properties of roasted chicken breast

MDA, a key product of lipid peroxidation, is commonly used as an indicator of lipid oxidation (39). A higher MDA level reflects more extensive oxidative degradation of lipids during high-temperature roasting, which may lead to flavor deterioration and reduced nutritional value. As shown in Figure 2E, dietary supplementation with DPSR and FDPSR significantly reduced the MDA content in roasted chicken breast, indicating enhanced lipid antioxidant capacity. Protein carbonyl content is an important indicator for assessing protein oxidation. As shown in Figure 2F, there were no significant differences in carbonyl content between the CK group and the DPSR/FDPSR groups after roasting, suggesting that dietary supplementation had no significant effect on protein carbonylation levels.

Total sulfhydryl (-SH) content is another commonly used indicator related to protein oxidation (40). As shown in Figure 2G, both the DPSR and FDPSR groups exhibited significantly lower total sulfhydryl contents than the CK group after roasting. In general, a decrease in sulfhydryl content may reflect oxidation of sulfhydryl groups in cysteine residues and the formation of disulfide bonds during thermal processing (41). Such changes may alter protein conformation and aggregation behavior in muscle systems (42, 43). Although the lower sulfhydryl content in the treated groups indicates a greater loss of measurable thiol groups, the overall oxidation-related results suggest that DPSR and FDPSR improved lipid oxidative stability, as reflected by the lower MDA content, whereas their effects on protein oxidation were more complex and marker-dependent.

### 3.6 Sensory evaluation of roasted chicken using E-nose and E-tongue

**Figure 3:**
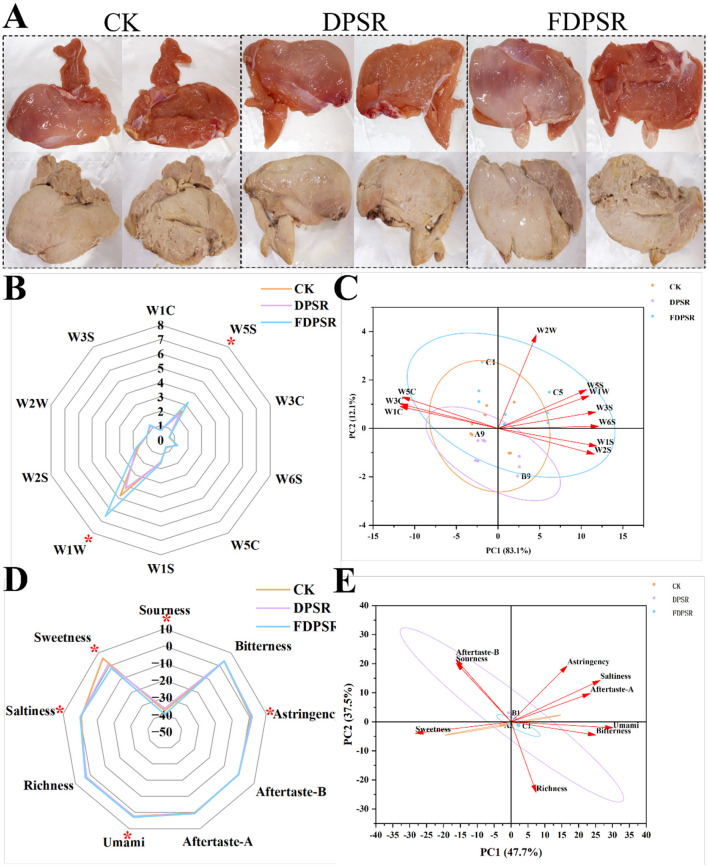
**(A)** Before-and-after photos of grilled chicken breast from the CK, DPSR, and FDPSR groups. **(B, C)** Radar plot and PCA (Principal Component Analysis) of the electronic nose. **(C, D)** Radar plot and PCA (Principal Component Analysis) of the electronic tongue.

Flavor is a key sensory attribute that significantly influences consumer preference. The E-nose and E-tongue are instrumental tools that simulate, to some extent, the human olfactory and gustatory systems, respectively, and allow rapid discrimination of volatile and non-volatile profiles in food samples (44, 45). Radar plots and principal component analysis (PCA) results for the E-nose and E-tongue data are presented in Figures 3B – E. The radar area of the E-nose differed among treatment groups after roasting, suggesting that dietary supplementation was associated with changes in the volatile profile of roasted chicken. Relatively strong responses were observed for the W5S and W1W sensors, indicating that compounds to which these sensors are sensitive contributed to the differentiation among groups. The FDPSR group showed the highest responses for these sensors. However, because W5S and W1W may respond to multiple classes of volatile compounds, including nitrogen- and sulfur-containing compounds, their signals should be interpreted as indicators of compositional differences rather than direct evidence of improved or deteriorated flavor quality. PCA of the E-nose data showed that PC1 and PC2 accounted for 83.1 and 12.1% of the total variance, respectively, explaining 95.2% of the overall variation among samples (46). The separation among groups in the PCA plot suggests that the volatile profiles of roasted chicken differed among dietary treatments. These differences were further interpreted in combination with GC-MS and E-tongue results rather than on the basis of E-nose sensor responses alone.

The E-tongue radar plot (Figure 3D) demonstrated differences in bitterness, astringency, umami, richness, and sweetness across the samples. Significant differences were observed in sourness, astringency, umami, saltiness, and sweetness. The FDPSR group showed the highest response values for umami and saltiness, suggesting a more complex flavor profile. According to Ivarsson et al. (47), umami and saltiness are key contributors to the characteristic flavor of chicken, and the elevated responses in the FDPSR group indicate that dietary FDPSR enhanced overall flavor perception. PCA of the E-tongue results (Figure 3E) showed that the first two principal components explained 85.2% of the total variance, indicating effective dimensionality reduction of the taste data. The scattered distribution of samples in the PCA plot reflects significant differences in taste attributes among the groups and demonstrates the E-tongue's capability to clearly distinguish roasted chicken breast with different dietary treatments.

### 3.7 LC-MS-based analysis of non-volatile differential metabolites

**Figure 4:**
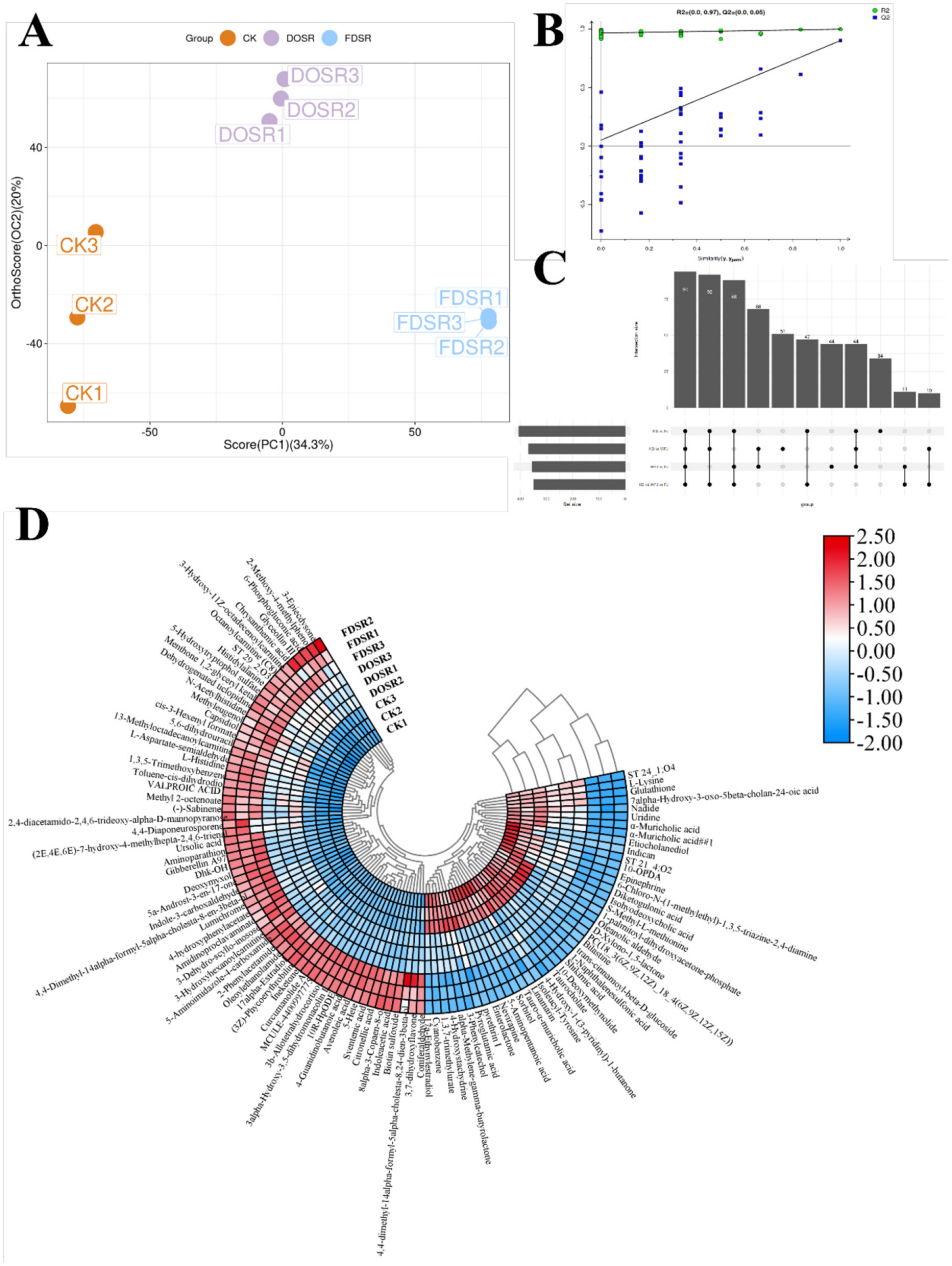
Analysis of LC-MS. **(A, B)** OPLS-DA. **(C)** Classification of substances. **(D)** Clustering heat map of all substances.

The metabolite profiles of roasted chicken breast from the CK, DPSR, and FDPSR groups were preliminarily analyzed. OPLS-DA was used as an exploratory multivariate approach to assess group-related variation. As shown in Figure 4A, an apparent tendency toward separation among the three groups was observed in the score plot, suggesting that dietary supplementation with DPSR and FDPSR was associated with differences in the metabolite profiles of roasted chicken breast. The model parameters (R^2^X = 0.0512, R^2^Y = 0.998, and Q^2^ = 0.877) provide supportive information for group discrimination under the present analytical conditions, but do not constitute definitive evidence of predictive robustness (Figure 4B).

A total of 1,267 secondary differential metabolites were identified based on secondary-level identification and filtered using predefined thresholds (*p* < 0.05 and VIP > 1.0). A Venn diagram revealed 94 shared differential metabolites across the CK, DPSR, and FDPSR groups (Figure 4C). Subsequently, 52 metabolites were selected using stricter criteria (*p* < 0.05, VIP > 1.7), and a hierarchical clustering heatmap was constructed (Figure 4D). These metabolites were mainly composed of amino acids and their derivatives, aromatic compounds, amides, ketones, heterocyclic compounds, lipids, and lipid-like molecules. Notably, the proportion of amino acids and their derivatives increased from 45.41% in the CK group to 48.37% and 46.77% in the DPSR and FDPSR groups, respectively. Methionine, histidine, and histidyl-alanine were the predominant contributors. Previous studies have shown that free amino acids and organic acids are key taste-active compounds in meat products (48). Ge et al. (49) reported that methionine injection enhanced protein synthesis and antioxidant capacity in broilers, which improved growth performance. These findings align with our antioxidant results and may partially explain the reduced oxidative damage in DPSR- and FDPSR-treated groups. In addition, methionine can undergo Maillard reactions with reducing sugars during roasting, and its sulfur-containing side chain may break down into sulfur volatiles, enhancing meaty and savory notes. Dietary histidine supplementation has also been shown to improve the nutritional and flavor quality of grass carp by modulating free amino acid and fatty acid profiles (50). In our study, the significant increase in histidine content—especially in the FDPSR group—may contribute to enhanced roasted, nutty, and caramel-like flavor attributes in chicken breast. Ketones are typically associated with creamy and fruity notes and contribute to the overall flavor of roasted chicken (51). For example, 5α-androst-3-en-17-one is known to enhance fatty and meaty aromas. Its level increased by 0.62% in the FDPSR group compared to the CK group, enriching the roasted chicken's flavor complexity. A total of 15 heterocyclic compounds were identified, accounting for 9.61, 8.50, and 10.42% of total metabolites in the CK, DPSR, and FDPSR groups, respectively. The increased abundance of these compounds in the FDPSR group may enhance aroma intensity and roasted flavor—consistent with findings by Li et al. (52), who reported that elevated heterocyclic compound levels improved umami and meaty characteristics.

Overall, supplementation with DPSR and FDPSR, particularly FDPSR, was associated with changes in the abundance of amino acids and derivatives, ketones, and heterocyclic compounds. These compositional differences may be related to treatment-associated variation in roasted chicken flavor profiles, although further sensory and functional validation is required.

### 3.8 Analysis of volatile organic compounds (VOCs) by HS-SPME-GC-MS

**Figure 5:**
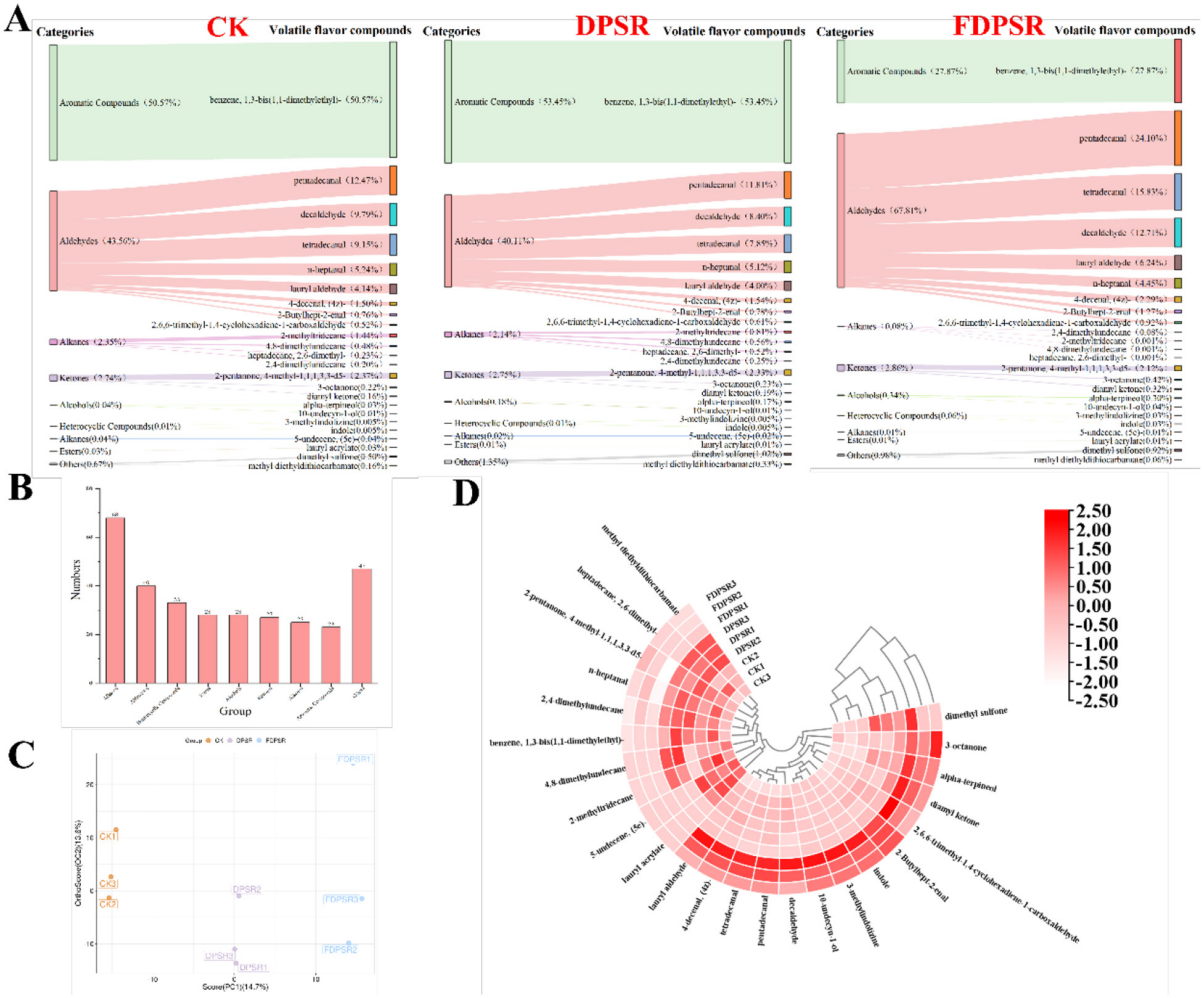
HS-SPME-GC-MS results analysis. **(A)** Differential metabolite content analysis (VIP > 1.0, *p* < 0.05). **(B)** Volatile compound composition statistics. **(C)** OPLS-DA. **(D)** Cluster heat map.

Volatile compounds in roasted chicken were analyzed using headspace solid-phase microextraction coupled with HS-SPME-GC-MS. Figure 5B summarizes the overall classification of volatile compounds detected across roasted chicken samples and does not by itself represent treatment-specific differences. Treatment-related differences were primarily reflected by the relative abundances of selected differential VOCs (Figure 5A, D, and Table 4). Consistent with previous studies (53), aldehydes, alcohols, and ketones were found to be the predominant volatile constituents in roasted chicken. These compounds are mainly derived from lipid oxidation and thermal degradation. Specifically, 40 aldehydes, 28 alcohols, and 27 ketones were detected. Aldehydes and alcohols are common volatiles in cooked meat products, such as hexanal, octanal, heptanal, and 1-octen-3-ol, and are considered major contributors to meat aroma (54). Although roasting may alter their concentrations, these compounds remain essential to the characteristic flavor profile of roasted chicken (55). Fatty aldehydes and alcohols are primarily formed from the oxidation of unsaturated fatty acids such as oleic acid, α-linolenic acid, linoleic acid, and arachidonic acid, yielding compounds like 1-pentanol, 1-hexanol, hexanal, 1-heptanol, heptanal, 1-octanol, octanal, benzaldehyde, and nonanal (56–58). These volatiles often impart species-specific meaty notes and are widely detected in various cooked meats, including pork, lamb, beef, crocodile, duck, and crab (59).

**Table 4:** Effect of dietary addition of DPSR and FDPSR on volatile compounds in chicken breast meat.

Name of compound	Retention time	Formula	CAS	Content		
				CK	DPSR	FDPSR
lauryl acrylate	1,893.84667	C15H28O2	2,156-97-0	153.99 ± 20.12^a^	34.71 ± 57.71^b^	1.39 ± 0.00^b^
3-methylindolizine	2,480.19333	C9H9N	1761-10-0	1.39 ± 0.00^b^	1.39 ± 0.00^b^	139.82 ± 47.60^a^
indole	2,418.44667	C8H7N	120-72-9	1.39 ± 0.00^b^	1.39 ± 0.00^b^	120.30 ± 35.11^a^
5-undecene, (5e)-	587.515	C11H22	764-97-6	179.50 ± 29.03^a^	80.26 ± 136.62^ab^	1.39 ± 0.00^b^
2,4-dimethylundecane	686.781778	C13H28	17,312-80-0	900.78 ± 212.00^a^	1,206.58 ± 144.10^a^	350.09 ± 210.49^b^
2-methyltridecane	965.602667	C14H30	1,560-96-9	6,584.29 ± 2551.50^a^	3,951.01 ± 3525.12^ab^	1.39 ± 0.00^b^
4,8-dimethylundecane	714.5202	C13H28	17,301-33-6	2,177.55 ± 597.97^a^	2,721.41 ± 2377.63^a^	1.39 ± 0.00^a^
heptadecane, 2,6-dimethyl-	953.909	C19H40	54,105-67-8	1,053.64 ± 1371.09^ab^	2,552.80 ± 274.21^a^	1.39 ± 0.00^b^
2-pentanone, 4-methyl-1,1,1,3,3-d5-	847.030444	C6H7D5O	4,840-81-7	10,800.42 ± 527.53^a^	11,370.13 ± 495.16^a^	9,107.79 ± 1167.55^b^
3-octanone	821.839	C8H16O	106-68-3	986.61 ± 199.32^b^	1,126.18 ± 246.60^b^	1,815.66 ± 703.56^a^
diamyl ketone	1,260.4825	C11H22O	927-49-1	735.34 ± 70.48^b^	916.00 ± 206.63^b^	1,371.90 ± 263.06^a^
2,6,6-trimethyl-1,4-cyclohexadiene-1-carboxaldehyde	1,717.45	C10H14O	162,376-82-1	2,363.54 ± 350.99^b^	2,953.85 ± 169.04^ab^	3,963.34 ± 1141.27^a^
2-Butylhept-2-enal	1,342.335	C11H20O	73,757-26-3	3,457.78 ± 257.35^b^	3,801.35 ± 318.66^b^	5,440.80 ± 79.67^a^
4-decenal, (4z)-	1,283.92375	C10H18O	2,1662-09-9	6,828.81 ± 8515.54^b^	7,515.52 ± 551.28^b^	9,859.72 ± 1253.14^a^
decaldehyde	1,220.029	C10H20O	112-31-2	44,716.80 ± 4257.44^ab^	40,990.29 ± 2751.82^b^	54,596.36 ± 7016.07^a^
lauryl aldehyde	1,527.90222	C12H24O	112-54-9	18,906.35 ± 2716.63^b^	19,539.47 ± 1031.91^b^	26,803.77 ± 4798.60^a^
n-heptanal	705.942	C7H14O	111-71-7	23,911.28 ± 1985.75^a^	24,986.49 ± 1288.75^a^	19,112.33 ± 2439.75^b^
pentadecanal	1,936.71889	C15H30O	2,765-11-9	56,936.11 ± 12984.80^b^	57,643.73 ± 7257.54^b^	103,572.16 ± 18668.39^a^
tetradecanal	1,806.93	C14H28O	124-25-4	41,779.03 ± 9017.92^b^	38,325.42 ± 3623.92^b^	68,022.18 ± 11963.21^a^
dimethyl sulfone	1,786.573	C2H6O2S	67-71-0	41,779.03 ± 9017.92^a^	38,325.42 ± 3623.92^a^	68,022.18 ± 11963.21^a^
methyl diethyldithiocarbamate	1,930.93,667	C6H13NS2	686-07-7	752.00 ± 86.39^b^	1,624.71 ± 56.22^a^	267.27 ± 153.29^c^
benzene, 1,3-bis(1,1-dimethylethyl)-	1,105.71667	C14H22	1,014-60-4	230,879.34 ± 66432.79^a^	260,853.90 ± 58442.20^a^	119,741.98 ± 19592.00^b^
10-undecyn-1-ol	2,262.53	C11H20O	2,774-84-7	1.39 ± 0.00^b^	1.39 ± 0.00^b^	151.20 ± 55.17^a^
alpha-terpineol	1,506.8075	C10H18O	98-55-5	169.90 ± 7.38^c^	890.17 ± 56.14^b^	1,298.51 ± 287.84^a^

Each the sample underwent three replications, and data were presented as mean values ± standard deviation. Different lowercase letters of superscript (a, b, c and ab, *p* < 0.05) denote levels of compounds with statistically significant differences determined by one-way ANOVA (Duncan's multiple comparison test).

Based on the VIP > 1.0 and *p* < 0.05 thresholds, 24 differential volatile metabolites were screened, comprising 8 aldehydes, 4 alkanes, 3 ketones, 2 alcohols, 2 heterocyclic compounds, 1 aromatic compound, 1 alkene, 1 ester, and 2 classified as others. Together, these volatiles accounted for over 95% of the total VOCs detected (Table 4). Notably, significant differences were observed in VOC profiles among the CK, DPSR, and FDPSR groups. Aldehydes, known for their low odor thresholds and high flavor impact (60), were present at markedly higher levels in the FDPSR group (67.81%) compared to CK (43.56%) and DPSR (40.11%; Figure 5A). Among these, decanal and tetradecanal were the most abundant, contributing intense meaty, fatty, and buttery notes, respectively (61, 62), indicating a substantial effect of FDPSR treatment on aroma development. In contrast, the CK and DPSR groups exhibited higher relative contents of aromatic compounds, which are known to enhance meaty and roasted flavors and may interact synergistically with other volatiles. Hydrocarbons, largely produced *via* autoxidation of long-chain fatty acids (63), were abundant across all groups. Although these compounds have high odor thresholds and contribute less directly to aroma (64), they are important structural components of the overall aroma system and play a stabilizing role (65). Under certain conditions, hydrocarbons may further oxidize to aldehydes and ketones, thereby indirectly influencing flavor development (66). The higher hydrocarbon content observed in the DPSR group (Figure 5A) may be a contributing factor to its enhanced flavor perception. Differences in VOC abundance among samples were further visualized in a heatmap (Figure 5D). Only one aromatic compound, 1,3-di-tert-butylbenzene, was identified across all groups. However, this compound is typically not considered a favorable aroma contributor in poultry, as it can intensify greasy off-notes and potentially mask desirable meaty, roasted, or fatty aromas.

To further clarify the key aroma contributors, relative odor activity values (ROAV) were calculated. Compounds with ROAV ≥ 1 were considered to significantly contribute to the overall aroma profile (67, 68). Six such key volatile compounds were identified, including two alkanes, one aromatic compound, one aldehyde, one ketone, and one organosulfur compound (Table 5). Among them, hexylbenzene, n-tridecane, and n-tetradecane were the primary aroma-active compounds in all three groups. Hexylbenzene, an aromatic compound, imparts a distinct floral-spicy character, while n-tridecane and n-tetradecane contribute to the fatty mouthfeel and overall flavor richness. Decanal, a typical lipid oxidation product, adds a buttery and fatty aroma, enhancing the sensory complexity of roasted meat (62). Overall, the volatile analysis suggests that dietary supplementation with DPSR and FDPSR, particularly FDPSR, was associated with changes in selected aroma-related compounds in roasted chicken.

**Table 5:** The ROAV of volatile flavor compounds in breast muscles of chicken.

Compound	Odor threshold (μg/kg)	ROAVs		
		CK	DPSR	FDPSR
ethylbenzene	0.000004	100.00	100.00	100.00
n-tridecane	0.00002	23.56	26.97	26.29
n-tetradecane	0.0011	9.95	9.03	6.42
decaldehyde	0.00002	3.96	6.37	6.37
dimethyl sulfone	0.0002	4.76	5.64	5.03
2-isooctanone	0.0003	0.00027	2.60	0.00019

ROAV≥1.

### 3.9 Correlation analysis between volatile and non-volatile flavor compounds and sensory attributes in chicken meat

**Figure 6:**
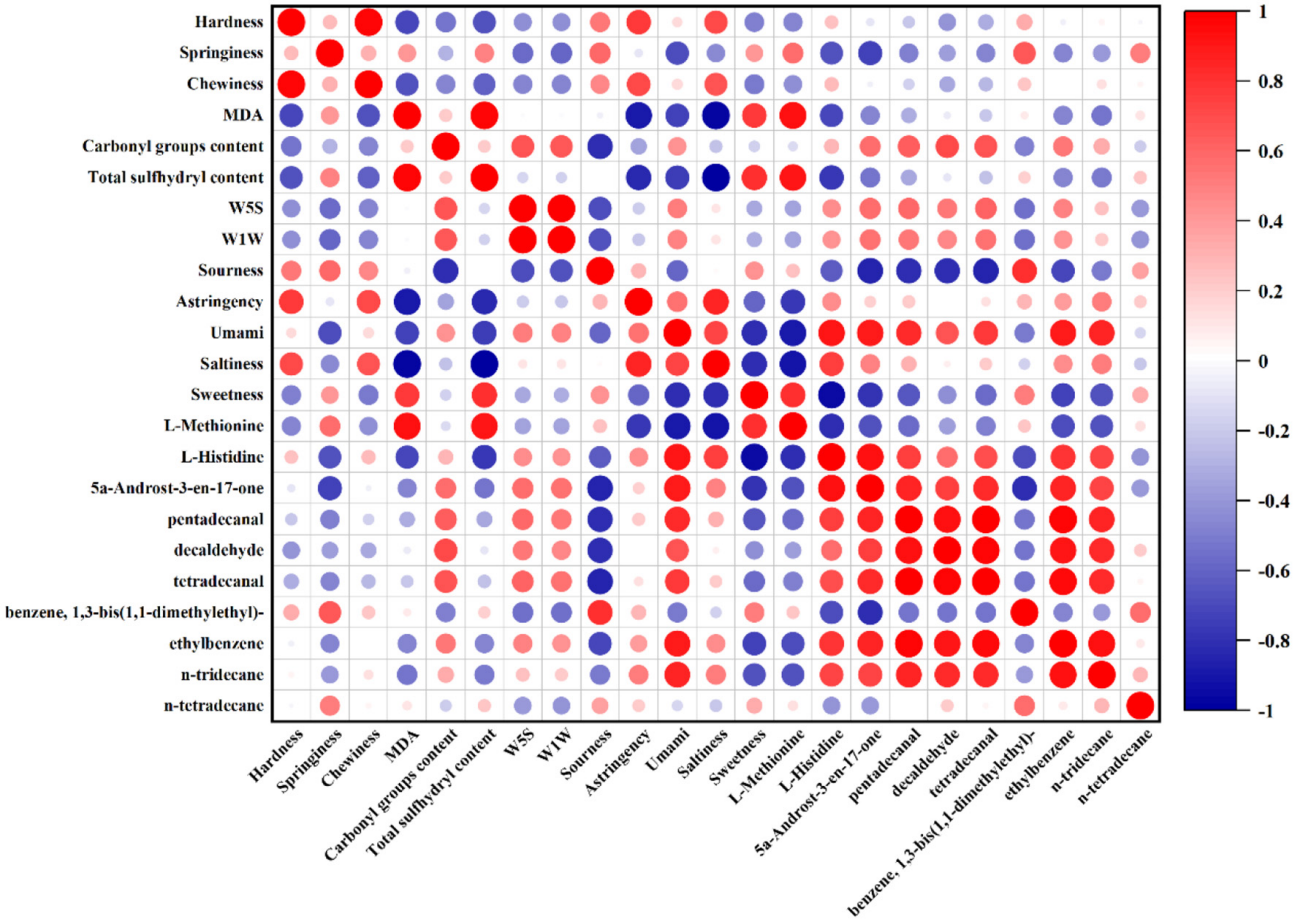
Correlation heatmap of the results of texture profile analysis (TPA), electronic nose, electronic tongue, key volatile and non-volatile metabolites, malondialdehyde, total sulfhydryl content and carbonyl groups content.

The relationship between meat texture and flavor is critical for understanding the sensory quality of roasted chicken. To investigate these interactions, a correlation analysis was conducted among texture profile analysis (TPA) parameters, E-nose and E-tongue responses, key volatile compounds, and oxidative indicators, as illustrated in Figure 6. The results revealed a significant negative correlation between hardness and total sulfhydryl content, indicating that a reduction in sulfhydryl groups corresponds to increased protein oxidation, aggregation, and muscle stiffness (43). Notably, umami perception showed strong positive correlations with several compounds, including histidine, 5α-androst-3-en-17-one, aldehydes, aromatic compounds, and alkanes, suggesting that these molecules play important roles in enhancing umami taste in roasted chicken breast. E-nose sensors W5S and W1W exhibited negative correlations with elasticity, sourness, and 1,3-di-tert-butylbenzene, but were positively correlated with carbonyl content, umami intensity, histidine, 5α-androst-3-en-17-one, pentadecanal, decanal, and tetradecanal. These findings indicate that W5S and W1W are responsive to compounds associated with lipid oxidation and flavor enhancement. MDA, a widely recognized marker of lipid oxidation (60), was found to be negatively correlated with hexylbenzene and n-tridecane levels. This suggests that these compounds may act as intermediate products during lipid oxidation and are progressively converted into MDA as oxidation advances. Furthermore, a significant positive correlation was observed between total sulfhydryl content and sweetness, implying that protein oxidation—and the resulting loss of sulfhydryl groups—may adversely affect sweet taste perception in roasted meat. The observed negative correlations between carbonyl content and several favorable flavor compounds further support the notion that oxidative degradation compromises meat flavor. Collectively, these correlations suggest that oxidative status, volatile compounds, non-volatile metabolites, and instrumental sensory responses may be interrelated in roasted chicken.

### 3.10 Molecular docking simulation analysis of potential odorant-receptor interactions

**Figure 7:**
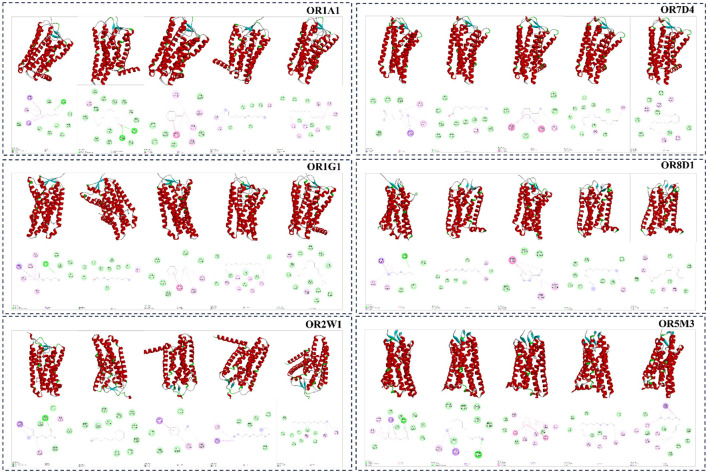
Simulation results for molecular docking between key differential volatile compounds (2-isooctanone, decanal, ethylbenzene, n-tetradecane, and n-tridecane) and olfactory receptors.

To further explore the possible interactions between representative aroma-active compounds and olfactory receptors, molecular docking simulation analysis was performed using five key volatile compounds −2-isooctanone, decanal, ethylbenzene, n-tetradecane, and n-tridecane—and six human olfactory receptors (OR1A1, OR1G1, OR2W1, OR5M3, OR7D4, and OR8D1) (69, 70). The binding positions and binding sites of five key volatile compounds with six human olfactory receptors are shown in Figure 7. The calculated binding energies are summarized in Table 6. All 30 ligand-receptor complexes showed binding energies below −4.0 kcal/mol, suggesting potentially favorable interactions under the simulation conditions (71, 72). The simulated binding energies ranged from −6.4 to −4.2 kcal/mol. Among the tested compounds, ethylbenzene showed the lowest average binding energy, whereas decanal showed the highest average binding energy. Different volatile compounds also exhibited different predicted interaction patterns with the selected receptors. For example, OR1A1 was predicted to interact with ligands through residues such as TYR258 and PHE206, whereas OR7D4 involved residues such as PHE521 and ALA108. In addition, some amino acid residues, including PHE251, were observed in more than one simulated receptor-ligand complex.

**Table 6:** Summary of the interaction forces between olfactory receptors and ligands.

Ligands	Reporter	Docking score (kcal/mol)	Hydrophobic interactions	Hydrogen bonds
2-isooctanone	OR1A1	−5.6	Val203	Tyr250
decaldehyde	OR1A1	−5.1	Leu262	Asn176, Tyr178
ethylbenzene	OR1A1	−6.0	Tyr258, Val203, Val254, Tyr276, Phe206	/
n-tetradecane	OR1A1	−5.1	Phe72, Trp149, Lys80	/
n-tridecane	OR1A1	−5.8	Ile105, Ile181, Tyr258, Val254, Phe206, Tyr276, Tyr250	/
2-isooctanone	OR1G1	−4.8	Ile142, Cys141, Pro138, Pro58	Thr57
decaldehyde	OR1G1	−4.5	Pro138	/
ethylbenzene	OR1G1	−5.2	Ala112, Phe251, Phe252	/
n-tetradecane	OR1G1	−5.1	Phe252, Phe256, Phe260, Tyr278, Val108, Phe104	/
n-tridecane	OR1G1	−4.5	Phe252, Phe260	/
2-isooctanone	OR2W1	−4.6	Ile216, Tyr220	Ser217
decaldehyde	OR2W1	−4.5	/	/
ethylbenzene	OR2W1	−5.0	Phe200, Pro182	/
n-tetradecane	OR2W1	−4.5	Leu274	/
n-tridecane	OR2W1	−5.1	Pro182, Pro262, Phe200	/
2-isooctanone	OR5M3	−5.4	Met197, Ile103, Tyr257, His107	His107
decaldehyde	OR5M3	−4.2	Val211	Tyr207
ethylbenzene	OR5M3	−6.4	Met197, Val106, Ile103, Ala201, His107, Leu200, Ala157	/
n-tetradecane	OR5M3	−5.1	Leu142, Phe145, Pro146, Ile214, Val211	/
n-tridecane	OR5M3	−5.0	Phe118, Tyr121, Ile214, Ala115, Pro146, Phe111	/
2-isooctanone	OR7D4	−4.5	Ala108, Phe251, Tyr278	/
decaldehyde	OR7D4	−4.5	Tyr252	/
ethylbenzene	OR7D4	−4.8	Ala108	/
n-tetradecane	OR7D4	−5.0	Ala108, Tyr252	/
n-tridecane	OR7D4	−4.7	Phe251, Tyr278, Ala108, Leu206, Phe155	/
2-isooctanone	OR8D1	−4.2	Phe205, Ile201, Ile202	Asn206
decaldehyde	OR8D1	−4.2	Phe257, Val273	/
ethylbenzene	OR8D1	−4.8	Phe260, Leu208, Phe200	/
n-tetradecane	OR8D1	−4.5	Val273	
n-tridecane	OR8D1	−4.6	Val273, Leu17, Leu30, Phe31	/

Overall, these docking simulation results suggest that the selected volatile compounds may differ in their potential binding preferences toward different olfactory receptors.

## 4 Discussion

### 4.1 Impact of DPSR and FDPSR on meat quality and flavor

This study showed that dietary supplementation with DPSR and FDPSR was associated with changes in the textural and sensory-related characteristics of roasted chicken. Compared with the CK group, the DPSR group showed lower hardness and chewiness together with higher springiness and adhesiveness, suggesting an improvement in textural acceptability. LF-NMR results indicated treatment-associated differences in water mobility after roasting, whereas SEM provided limited morphological observations suggesting only subtle variation in muscle microstructure among treatments. Therefore, although changes in water distribution may be related to the observed differences in texture, the relationship among microstructure, water mobility, and tenderness requires further clarification. From a practical perspective, the textural changes observed in the DPSR and FDPSR groups remain relevant, because texture is a key quality attribute influencing consumer preference in poultry products (73). Under the present experimental conditions, the results suggest that DPSR and FDPSR may modulate roasted chicken texture, but the underlying structural and water-related mechanisms should be interpreted cautiously and require further validation.

### 4.2 Antioxidant effects of DPSR and FDPSR

The oxidation-related results suggest that dietary supplementation with DPSR and FDPSR improved lipid oxidative stability, as evidenced by the lower MDA content observed after roasting. However, the effects on protein oxidation-related indicators were less straightforward. Although both DPSR and FDPSR groups showed lower total sulfhydryl contents than the CK group, protein carbonyl content did not differ significantly among treatments. These findings indicate that the effects of dietary supplementation on oxidation-related parameters were marker-dependent, with clearer evidence for reduced lipid oxidation than for protein oxidation.

### 4.3 Flavor enhancement and the role of metabolites

The metabolomics and volatile analyses indicate that dietary supplementation with DPSR and FDPSR, particularly FDPSR, was associated with differences in flavor-related compounds in roasted chicken. In the metabolomics dataset, amino acids and their derivatives represented the major class of differential metabolites, with methionine, histidine, and histidyl-alanine among the predominant contributors. These compounds are closely related to meat flavor formation, because amino acids can directly contribute to taste and also serve as important precursors of aroma-active compounds generated during thermal processing (74). In parallel, volatile analysis showed that aldehydes were the dominant differential VOC class and were present at a markedly higher proportion in the FDPSR group than in the CK and DPSR groups, with decanal and tetradecanal being particularly abundant. Given their low odor thresholds and known contributions to fatty and meaty notes, these compounds may be involved in the flavor-related differences observed among treatments. Consistently, the E-tongue results showed stronger umami- and saltiness-related instrumental responses in the FDPSR group, further supporting a treatment-associated shift in flavor-related profiles. Taken together, these results suggest that FDPSR was more strongly associated than DPSR with changes in both non-volatile and volatile compounds linked to roasted chicken flavor.

### 4.4 Correlations between texture, flavor, and oxidative markers

The correlation analysis provides exploratory information on the relationships among texture, oxidation-related parameters, flavor-related compounds, and instrumental sensory responses. The negative correlation between hardness and total sulfhydryl content suggests that changes in protein oxidation-related indicators may be associated with textural variation. Likewise, the positive correlations between umami-related responses and compounds such as histidine, 5α-androst-3-en-17-one, aldehydes, and aromatic compounds suggest that these molecules may be involved in the observed flavor-related differences.

### 4.5 Molecular docking simulation and potential odorant-receptor interactions

The molecular docking simulation analysis in the present study was used as a complementary *in silico* approach to explore the possible interactions between selected volatile compounds and olfactory receptors. The results suggested that different aroma-active compounds may show different predicted binding affinities and binding patterns toward the selected receptors. In particular, ethylbenzene showed relatively lower binding energy values than several other compounds, whereas decanal showed comparatively weaker predicted binding affinity under the simulation conditions. In addition, some amino acid residues, such as PHE251, were involved in more than one simulated receptor-ligand complex, suggesting that these residues may participate in the recognition of multiple volatile compounds.

## 5 Conclusion

In summary, dietary supplementation with *Dendrobium devonianum* Paxt stem residue (DPSR) and its fermented form (FDPSR) was associated with changes in roasted chicken quality and flavor-related characteristics. DPSR was more closely associated with improvements in selected textural attributes, whereas FDPSR showed a stronger association with flavor-related metabolites, volatile compounds, and instrumental sensory responses. Both treatments reduced lipid oxidation after roasting, indicating improved oxidative stability of roasted chicken breast. In addition, metabolomics and volatile analyses revealed treatment-related differences in amino acids, aldehydes, ketones, and heterocyclic compounds, suggesting that DPSR and especially FDPSR may modulate the flavor-related chemical profile of roasted chicken. In terms of growth performance, DPSR and FDPSR affected broiler growth efficiency during the starter phase, but no significant differences were observed among treatments over the overall 1–45 d feeding period. Collectively, these results indicate that DPSR and FDPSR, particularly FDPSR, have potential as sustainable feed ingredients for modulating roasted chicken quality and flavor-related traits.

Several limitations of this study should also be acknowledged. First, although 63 broilers were included in the feeding trial, only one representative bird from each replicate pen was selected for postmortem meat quality and omics analyses, resulting in three biological replicates per treatment group. Second, SEM provided morphological observations only and did not directly assess the internal structural basis of water retention. Third, no receptor-based validation assays or trained human sensory panel evaluation were conducted. Finally, because gut microbiota profiling was not included in the present study, the possible contribution of intestinal microbial regulation to the observed effects of DPSR and FDPSR remains unclear. Overall, the present results suggest that DPSR and FDPSR have potential as sustainable feed ingredients for modulating roasted chicken quality and flavor, but future studies should incorporate larger sample sizes, direct water-holding and structural validation, gut microbiota analysis, receptor-based assays, and trained human sensory evaluation to further confirm the biological relevance, sensory significance, and mechanistic basis of these findings.

## Data Availability

The data are deposited in the China National Center for Bioinformation repository under accession number PRJCA060249 (https://ngdc.cncb.ac.cn/bioproject/browse/PRJCA060249).
